# Risk evaluation of splenic hilar or splenic artery lymph node metastasis and survival analysis for patients with proximal gastric cancer after curative gastrectomy: a retrospective study

**DOI:** 10.1186/s12885-019-6112-4

**Published:** 2019-09-11

**Authors:** Peng Ding, Ziming Gao, Chen Zheng, Junqing Chen, Kai Li, Shan Gao

**Affiliations:** 1 Department of Surgical Oncology and General Surgery, The First Affiliated Hospital of China Medical University, Shenyang, 110001 China; 2 Department of Gynecology and Obstetrics, Shengjing Hospital of China Medical University, Sanhao Street 36, Shenyang, 110001 China

**Keywords:** Metastasis, Prognosis, Proximal gastric cancer, Splenic hilar lymph node

## Abstract

**Background:**

As splenectomy and spleen-preserving lymphadenectomy are performed only in some proximal gastric cancer patients, it is difficult to identify patients who have undergone radical gastrectomy with or without splenic hilar (No.10) or splenic artery (No.11) lymph node metastases. We aimed to determine the risk factors for No.10 and No.11 lymph node metastases and evaluate the survival significance of No.10 and No.11 lymph node dissection in advanced proximal gastric cancer patients.

**Methods:**

A total of 873 advanced proximal gastric cancer patients who underwent curative gastrectomy with or without splenectomy or pancreaticosplenectomy were analyzed retrospectively. The clinicopathological characteristics of 152 patients who underwent splenectomy or pancreaticosplenectomy were analyzed to determine the risk factors for No.10 and No.11 lymph node metastases. The survival difference between patients with No.10 and No.11 lymph node dissections and those who did not undergo these dissections were compared.

**Results:**

Patients with No.10 and No.11 lymph node metastases had very poor prognoses. Tumor invasion of the greater curvature and No.2 and No.4 lymph node metastases were independent risk factors for No.10 and No.11 lymph node metastases. No survival differences were evident between patients with No.10 and No.11 lymph node metastases who underwent No.10 and No.11 lymph node dissections and those who did not undergo these dissections but were at high risks of No.10 and No.11 lymph node metastases.

**Conclusions:**

Splenic hilar or splenic artery lymph node dissection was not associated with increased survival, in proximal gastric cancer patients without direct cancer invasion of the spleen and pancreas, regardless of whether splenectomy, pancreaticosplenectomy, or spleen-preserving lymphadenectomy was performed.

## Background

Gastric cancer is a significant public health problem worldwide because it causes considerable morbidity and mortality [1]. Although the incidence of gastric cancer has decreased overall, that of proximal gastric cancer (PGC) has increased over the last few decades [2, 3]. PGC is a tumor located within the proximal third of the stomach, which includes the gastric cardia, esophageal-gastric junction, and fundus. Compared to distal gastric cancers, PGCs have worse prognoses because they are more aggressive and tend to be advanced by the time of diagnosis [4, 5].

Resection of the primary lesion with dissection of the D2 lymph nodes (LNs) is the only effective treatment for PGC. It is common to resect the spleen and/or the tail of the pancreas when gastrectomy is performed to completely remove the splenic hilar (No.10) and splenic artery (No.11) LNs. Because many studies have shown that splenectomy or pancreaticosplenectomy has higher postoperative complications and may not improve long-term survival, some researchers favor spleen-preserving lymphadenectomy for advanced proximal gastric cancer (APGC) [6–9]. Although controversy exists regarding the approach of No.10 and No.11 lymphadenectomy, there is no doubt that patients with splenic hilar or splenic artery LN metastases (SLNMs) have very poor prognoses [10, 11]. Given the high rates of operative complications during No.10 and No.11 LN dissections involving splenectomy or pancreaticosplenectomy, and the difficulties and non-completion rates associated with No.10 and No.11 LN dissections with spleen preservation, No.10 and No.11 LN dissections are not performed on all APGC patients. It is unclear whether APGC patients have increased survival rates after splenic hilar or splenic artery lymphadenectomy due to the difficulties in predicting SLNM preoperatively or intraoperatively.

The aim of this study was to determine the risk factors associated with SLNM by evaluating the clinicopathological characteristics relevant to SLNM in APGC patients who underwent curative gastrectomy with splenectomy, pancreaticosplenectomy, or spleen-preserving lymphadenectomy, and to clarify the prognostic significance of splenic hilar or splenic artery lymph node dissection in all patients with APCG.

## Methods

### Patients

Between January 1980 and October 2012, 1424 consecutive patients were histologically diagnosed with APGC and underwent radical proximal or total gastrectomy with D1^+^/D2 lymphadenectomyat the First Affiliated Hospital of China Medical University. The exclusion criteria were as follows: (1) patients with early gastric cancer, distant metastases, peritoneal dissemination, and direct invasion of the spleen or pancreas; (2) patients who died of postoperative complications or non-tumor-related events within 5 years of surgery; (3)patients who underwent chemotherapy or radiotherapy preoperatively; (4) patients with carcinomas in the remnant stomach; and (5) patients who were lost to follow-up. Consequently, 837 patients were included in the final analyses. In this study, the pathological tumor stage was evaluated using the 8th edition of the TNM-Union for International Cancer Control/AJCC classification.

### Surgical approach and postoperative adjuvant therapy

During the early 1980s, we performed standard D2 lymphadenectomy and removed the spleen and distal pancreas. However, given the high operative mortality and morbidity rates, after the mid-1980s, we preserved the spleen and pancreas when possible, except in cases when the tumor directly invaded the spleen or pancreas, the tumor was > 5 cm in the greater curvature, or the tumor had invaded the serosa and measured > 10 cm. When swollen LNs were present near the splenic hilum or splenic artery, combined resections of the spleen and pancreas or spleen-preserving lymphadenectomy were considered.

Therefore, according to the resection state of the spleen and No.10/No.11 LNs, we divided the 837 PGC patients who underwent gastrectomy into three groups: CR group (combined resections of the spleen and pancreas), SPR group (spleen-preserving resection with removal of No.10/No.11), and NSR group (spleen-preserving resection without removal of the No.10/No.11 LNs). Lymphadenectomy in the CR and SPR groups involved removal of No.1–7, No.8a, No.9, No.10, No.11p, No.11d, and No.12a LNs. Lymphadenectomy in the NSR group involved removal of No.1–7,No.8a, No.9, and No.12a LNs.

Postoperative chemotherapy regimens included 5-fluorouracil (5-Fu) plus cisplatin, 5-Fu plus mitomycin and epirubicin, 5-Fuplusleucovorin and cisplatin, 5-Fu plus cisplatin and epirubicin, or cisplatin plus oxaliplatin.

### Follow-up

All patients underwent standardized follow-up assessments every 3 months for the first 2 years postoperatively, every 6 months during the third postoperative year, and yearly thereafter. The follow-up interval spanned from the time of surgery until the patient’s death or for 5–10 years after surgery. Patients who lived for > 5 years or died of non-cancer-related events were considered to have survived.

### Statistical analysis

The chi-squared test was used to compare categorical variables. The Kaplan-Meier method was used for univariate survival analysis, and survival data were compared using the log-rank test. Multivariate analyses were performed using Cox proportional hazards models. All statistical analyses were conducted using IBM® SPSS® software version 22.0 (IBM Corporation, Armonk, NY, USA). A two-sided value of *P* < 0.05 was considered statistically significant.

## Results

### Patients’ characteristics and types of surgery

Of the APGC patients analyzed, 502 (60.0%) underwent total gastrectomy and 335 (40.0%) underwent proximal gastrectomy. Of these, 152 patients (18.2%) were in the CR group, 113 patients (16.5%) were in the SPR group, and 572 patients (83.5%) were in the NSR group. As shown in Table 1, patients who underwent CR or SPR had larger tumors, greater curvature involvement, serosal involvement, LN metastases, and later stage tumors.

**Table 1:** Clinicopathological findings of 837 patients with advanced proximal gastric cancer

	N	NSR (*n* = 572)	CR (*n* = 152)	SPR (*n* = 113)	*P*
Gender					0.947
Male	668	457 (68.4%)	120 (18.0%)	91 (13.6%)	
Female	169	115 (68.0%)	32 (18.9%)	22(13.0%)	
Age					0.387
< 60 years	460	306 (66.5%)	86 (18.7%)	68 (14.8%)	
≥ 60 years	377	266 (70.6%)	66 (17.5%)	45 (11.9%)	
Tumor size					< 0.001
< 5 cm	358	264 (73.7%)	38 (10.6%)	56 (15.6%)	
≥ 5 cm	479	308(64.3%)	114 (23.8%)	57 (11.9%)	
Gastrectomy type					0.106
Proximal	335	244 (72.8%)	52 (15.5%)	39 (11.6%)	
Total	502	328 (65.3%)	100 (19.9%)	74 (14.7%)	
Greater curvature involved					< 0.001
No	617	445 (72.1%)	91 (14.7%)	81 (13.1%)	
Yes	220	127 (57.7%)	61 (27.7%)	32 (14.5%)	
pT stage					< 0.001
pT2	96	75 (78.1%)	8 (8.3%)	13 (13.5%)	
pT3	282	212 (75.2%)	28 (9.9%)	42 (14.9%)	
pT4a	459	285 (62.1%)	116 (25.3%)	58 (12.6%)	
pN stage					0.003
pN0	266	198 (74.4%)	42 (15.8%)	26 (9.8%)	
pN1	149	106 (71.1%)	31(20.8%)	12 (8.1%)	
pN2	180	122 (67.8%)	26 (14.4%)	32 (17.8%)	
pN3a-3b	242	146 (60.3%)	53 (21.9%)	43 (17.8%)	
pTNM stage					< 0.001
IB-IIA	168	138 (82.1%)	12 (7.1%)	18 (10.7%)	
IIB-IIIA	432	293 (67.8%)	87 (20.1%)	52 (12.0%)	
IIIB-IIIC	237	141 (59.5%)	53 (22.4%)	43 (18.1%)	
Bormann type					0.475
Bor 1–2	160	107 (66.9%)	34 (21.3%)	19 (11.9%)	
Bor 3–4	677	465 (68.7%)	118 (17.4%)	94 (13.9%)	

*NSR*: Nosplenic hilar orsplenic arteryLN resection, *CR*: combination resectionof the stomach and spleen or spleen-co-pancreas, *SPR*: spleen-preserved No. 10/11 LNs removal

### Prognostic impact of No.10/No.11 LN metastases on APGC patients who underwent splenic hilar or splenic artery lymphadenectomy

**Fig. 1:**
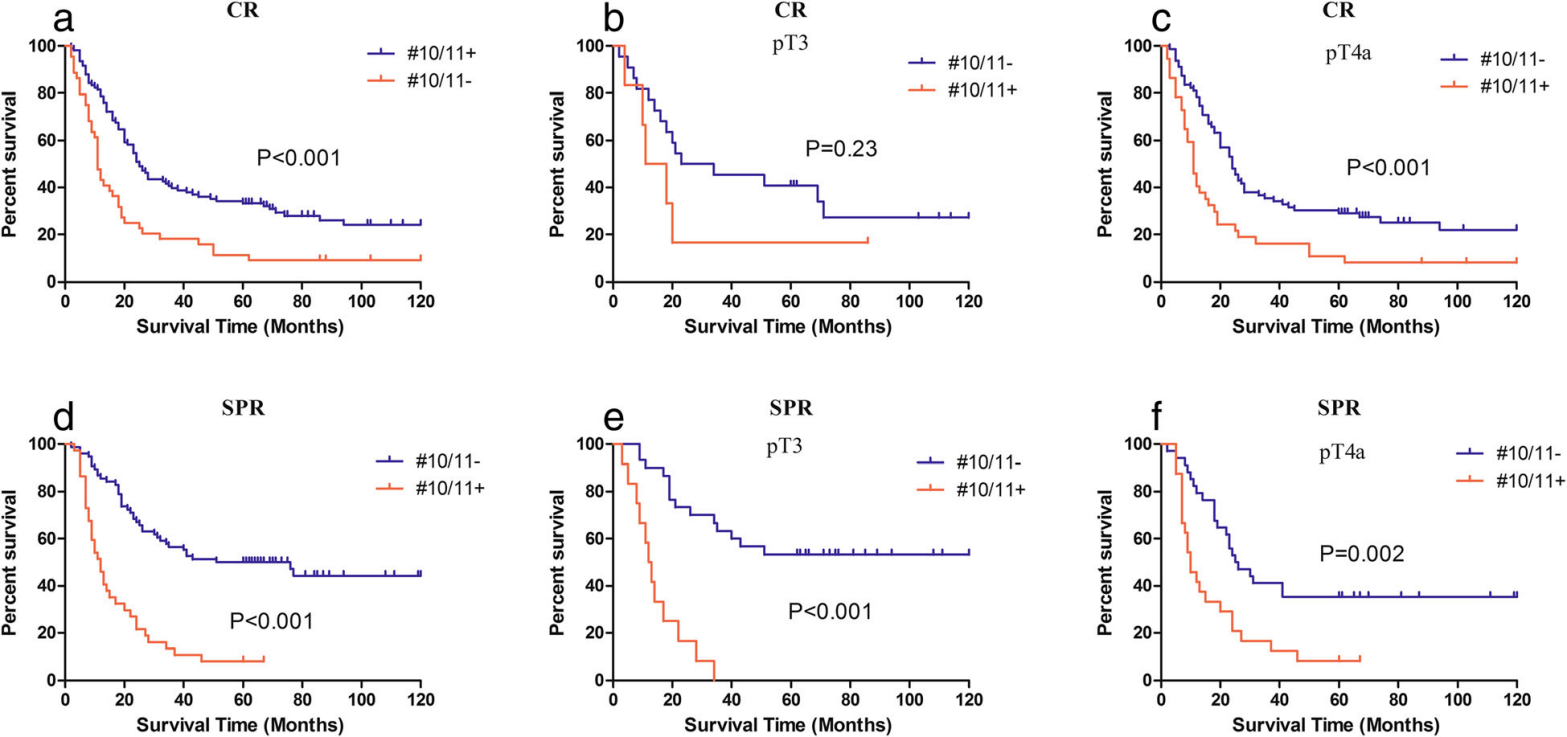
Kaplan–Meier plots of survival according to SLNM in patients with CR (**a**-**c**), and in patients with SPR (**d**-**f**)

Of the 152 patients who underwent CRs, 23.0% had positive No.10 LN, 16.4% had positive No.11 LN, and 27.8% had positive No.10/11 (No.10+/11+, SLNM) LNs. Of the 113 patients who underwent SPR, 6.2% had positive No.10, 27.4% had positive No.11, and 32.7% had No.10^+^/11^+^ LNs. In the CR group, 5-year survival rates of patients with No.10+/11+ LNs were less than those of patients who had No.10^−^/11^−^LNs(11.4 vs. 33.3%, *P* < 0.001) (Fig. 1a-c). Similarly, in the SPR group, patients who had No.10^+^/11^+^ LNs had worse prognoses than those who hadNo.10^−^/11^−^LNs (5-year survival rates: 8.1 vs. 50.0%, *P* < 0.001) (Fig. 1d-f). Multivariate Cox regression analyses showed that the presence of No.10^+^/11^+^ LNs was an independent risk factor for poor prognosis of CR and SPR patients who underwent No.10/No.11 lymphadenectomy (hazard ratio 1.745; 95% confidence interval 1.241–2.454; *P* = 0.001) (Table 2).

**Table 2:** Univariate and multivariate survival analysis of 265 APGC patients who underwentNo. 10/11 LNs removal (CR + SPR)

	Univariate analysis		Multivariate analysis		
	5-year survival rate	*P*	RR	95%CI	*P*
Gender					
Male	31.8%				
Female	27.8%	0.564			
Age					
< 60 years	31.2%				
≥ 60 years	30.6%	0.843			
Tumor size					
< 5 cm	41.5%				
5-10 cm	26.3%		1.467	1.049–2.051	0.025
≥ 10 cm	21.1%	0.001	1.515	0.969–2.369	0.069
Gastrectomy type					
Proximal	37.4%				
Total	27.6%	0.047	1.008	0.731–1.391	0.961
pT stage					
pT2	71.4%				
pT3	37.1%		1.399	0.657–2.980	0.384
pT4a	23.6%	< 0.001	1.860	0.904–3.826	0.092
pN stage					
pN0	51.5%				
pN1	41.9%		0.983	0.589–1.642	0.949
pN2	36.2%		1.048	0.648–1.694	0.849
pN3	8.3%	< 0.001	2.134	1.319–3.453	0.002
Bormann type					
Bor 1–2	43.4%				
Bor 3–4	27.8%	0.007	1.142	0.766–1.704	0.515
Differentiation					
Well	36.0%				
Poor	27.2%	0.025	1.164	0.862–1.572	0.323
Approach of lymphadenectomy					
SPR	36.3%				
CR	27.0%	0.058			
NO.10/11 lymphnode					
Negative	40.2%				
Positive	9.9%	< 0.001	1.745	1.241–2.454	0.001

*CR*: combination resectionof the stomach and spleen or spleen-co-pancreas; *SPR*: spleen-preserved NO.10/11 lymphadenectomy

### Relationships between No.10/11 LN metastases and the clinicopathological factors of patients who underwent CR of the spleen and pancreas

As shown in Table 3, No.10^+^/11^+^ LNs were closely associated with metastases of No.1/3(*P* = 0.002), No.2/4(*P* < 0.001), No.6(*P* = 0.034), No.7(*P* < 0.001), No.8(*P* = 0.006), and No.9(*P* < 0.001) LNs, poorly differentiated tumors(*P* = 0.016), and tumor invasions of the greater curvature of the stomach (*P* < 0.001). Logistic regression analyses determined that No.2/4 LN metastases(*P* = 0.030) and tumor invasion of the greater curvature(*P* < 0.001) were independent risk factors for SLNM (Table 3).

**Table 3:** Univariate and multivariate analysis of No.10/11LN metastasis for 152 advanced proximal gastric cancer patients who underwent curative gastrectomy with splenectomy or pancreatectomy

	Univariate analysis			Multivariate analysis		
	No.10/11 -	No.10/11+	*P* ^a^	RR	95%CI	*P* ^b^
No.1/3						
-	91 (77.1%)	27 (22.9%)				
+	17 (50.0%)	17 (55.0%)	0.002	1.162	0.374–3.616	0.795
No.2/4						
-	84 (89.4%)	10 (10.6%)				
+	24 (41.4%)	34 (58.6%)	< 0.001	6.780	2.316–19.846	< 0.001
No. 5						
-	97 (72.9%)	36 (27.1%)				
+	11 (57.9%)	8 (42.1%)	0.176			
No.6						
-	94 (74.6%)	32 (25.4%)				
+	14 (53.8%)	12 (46.2%)	0.034	2.615	0.735–9.306	0.138
No.7						
-	93 (78.8%)	25 (21.2%)				
+	15 (44.1%)	19 (55.9%)	< 0.001	2.483	0.811–7.602	0.111
No.8						
-	99 (75.0%)	33 (25.0%)				
+	9 (45.0%)	11 (55.0%)	0.006	1.869	0.469–7.454	0.376
No. 9						
-	106 (75.2%)	35 (24.8%)				
+	2 (18.2%)	9 (81.8%)	< 0.001	3.338	1.249–3.595	0.202
No. 12a						
-	100 (71.4%)	40 (28.6%)				
+	8 (66.7%)	4 (33.3%)	0.727			
Tumor size						
< 5 cm	27 (71.1%)	11 (28.9%)				
5-10 cm	64 (72.7%)	24 (27.3%)				
≥ 10 cm	17 (65.4%)	9 (34.6%)	0.769			
Bormann type						
Bor 1–2	28 (82.4%)	6 (17.6%)				
Bor 3	68 (73.1%)	25 (26.9%)		1.117	0.318–3.919	0.863
Bor 4	12 (48.0%)	13 (52.0%)	0.013	2.126	0.417–10.846	0.365
Serosa involved						
No	29 (80.6%)	7 (19.4%)				
Yes	79 (68.1%)	37 (31.9%)	0.150			
Differentiation						
Well	55 (80.9%)	13 (19.1%)				
Poor	53 (63.1%)	31 (36.9%)	0.016	1.313	0.465–3.707	0.607
Greater curvature involved						
No	75 (82.4%)	16 (17.6%)				
Yes	33 (54.1%)	28 (45.9%)	< 0.001	2.963	1.112–7.897	0.030

a:χ^2^test, b:Logistic regression method used on variables identified as significant by univariate analysis

+, positive for the lymph node metastasis; −, negative for the lymph node metastasis

Consequently, we made a risk classification of SLNM. Patients with No.2/4 LN metastases and tumors that invaded the greater curvature of the stomach were considered to have a high level of risk for SLNM. Patients with either No.2/4 LN metastases or tumors that had invaded the greater curvature were considered to have a midlevel risk for SLNM. Patients without No.2/4 LN metastases or tumors that had invaded the greater curvature were considered to have a low level of risk for SLNM.

### Clinicopathological and survival features of SLNM risk grade in patients with NSR

**Fig. 2:**
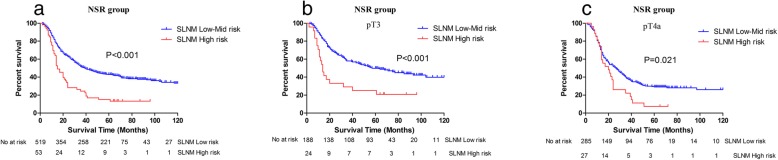
Kaplan–Meier plots of survival according to the risk grade of SLNM in the NSR group

Of the 572 patients in the NSR group, 53 (9.3%) had a high level of risk for SLNM and 519 (90.7%) had a low to midlevel risk for SLNM. In the NSR group, females and patients with higher pathological tumor-node-metastasis (pTNM) stages were at a higher risk of SLNM (Table 4). Patients with high SLNM risk had worse prognoses than those who had low to midlevel risk (5-year survival rates: 15.1 vs. 42.9%, *P* < 0.001), especially in pT3 (5-year survival rates: 25.0 vs. 49.9%, *P* < 0.001)and pT4a patients (5-year survival rates: 11.1 vs. 29.1%, *P* < 0.001) (Fig. 2).

**Table 4:** Comparison of the clinicopathological data in NSR according toSLNM risk grade

	SLNM low-mid risk n = (519)	SLNM high risk n = (53)	*P*
Gender			0.008
Male	422(92.3%)	35(7.7%)	
Female	97(84.3%)	18(15.7%)	
Age			0.103
< 60 years	272(88.9%)	34(11.1%)	
≥ 60 years	247(92.9%)	19(7.1%)	
Tumor size			0.672
< 5 cm	241(91.3%)	23(8.7%)	
≥ 5 cm	278(90.3%)	30(9.7%)	
Differentiation			0.873
Well	241(90.9%)	24(9.1%)	
Poor	278(90.6%)	29(9.4%)	
Bormann type			0.148
Bor 1–2	101(94.4%)	6(5.6%)	
Bor 3–4	418(89.9%)	47(10.1%)	
pT stage			0.084
pT2	73(97.3%)	2(2.7%)	
pT3	188(88.7%)	24(11.3%)	
pT4a	258(90.5%)	27(9.5%)	
pTNM stage			< 0.001
IB-IIA	138(100%)		
IIB-IIIA	274(93.5%)	19(6.5%)	
IIIB-IIIC	107(75.9%)	34(24.1%)	

*NSR*: Nosplenic hilar orsplenic arteryLN resection; *SLNM*:splenic hilar or splenic artery LN metastases

### Prognostic impact of the lymphadenectomy approach on patients with No.10/11 LN metastases

**Fig. 3:**
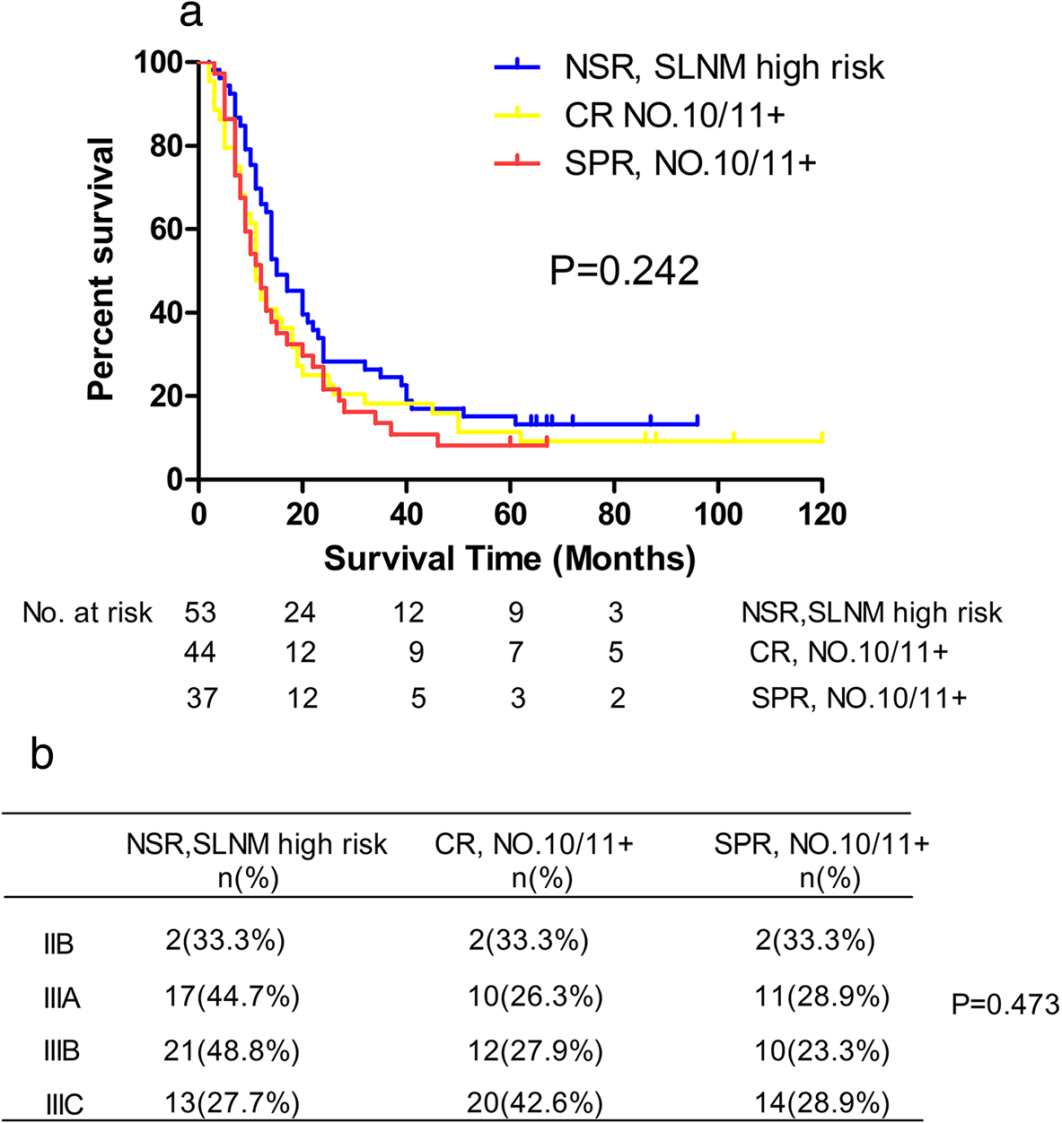
Survival comparison between NSR patients with high risk of SLNM, CR patients with No.10/11+ and SPR patients with No.10/11+ (**a**); the relationship of pTNM stages and NSR patients with high risk of SLNM, CR patients with No.10/11+ and SPR patients with No.10/11+ (**b**)

**Fig. 4:**
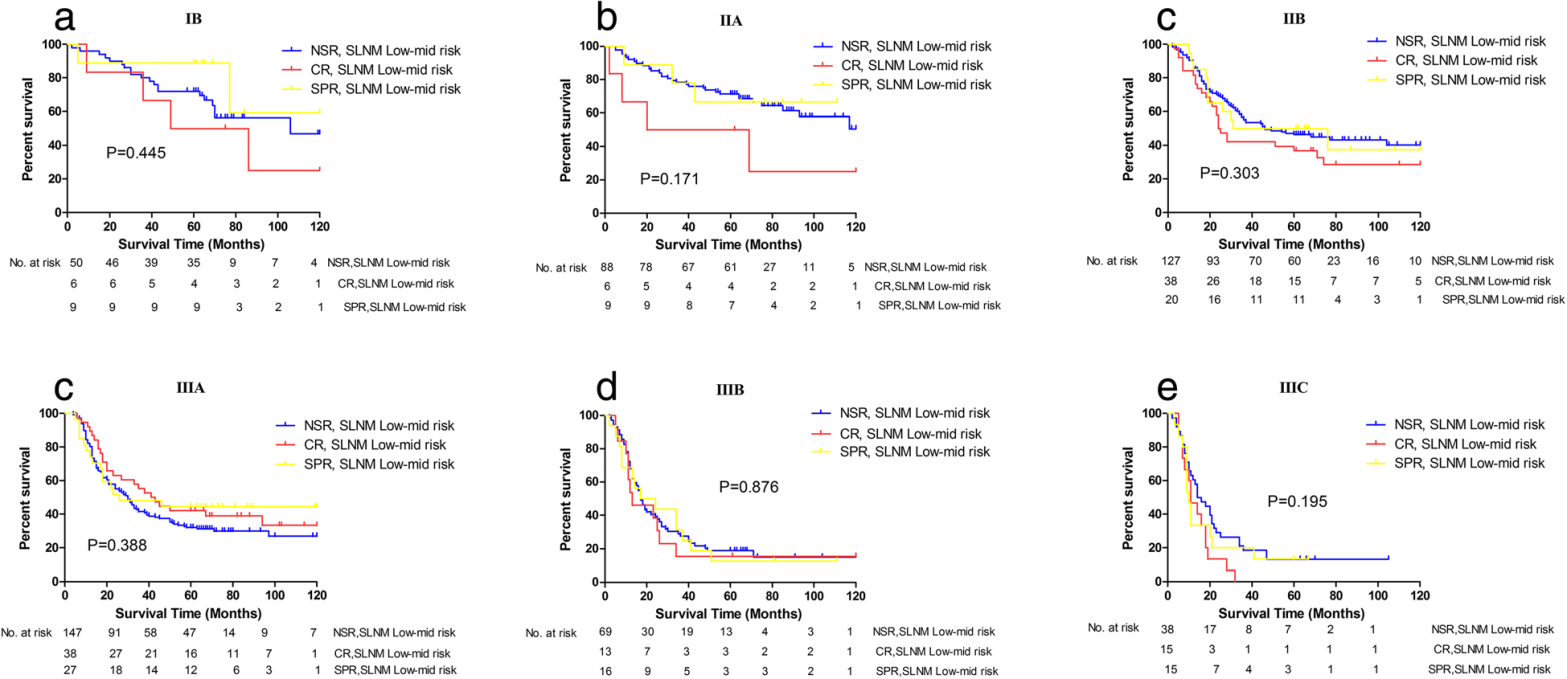
Survival comparison between NSR patients with low-mid risk of SLNM, CR patients with low-mid risk of SLNM and SPR patients with low-mid risk of SLNM

As shown in Fig. 3a, no significant differences in survival existed between the NSR group with high risk of SLNM and No.10^+^/11^+^LN patients of the CR or SPR groups (*P* = 0.242). Thus, regardless of whether spleen-preserving surgery was performed, lymphadenectomy approach did not affect the prognoses of patients with No.10^+^/11^+^ LNs. Considering patients’ distribution, no significant bias existed from pTNM stages (*P* = 0.473) (Fig. 3b). In addition, for the patients with low to midlevel risk of SLNM, there was no difference in survival between the NSR, CR, and SPR groups in the same TNM stage (Fig. 4).

### Operative features and postoperative complications in the NSR, CR, and SPR groups

The patients with NSR and SPR had a shorter operation time, lower incidence of perioperative transfusion, shorter hospital stay, and lower incidence of non-lethal surgical complication than patients within the CR group (Table 5).

**Table 5:** The features of operation and postoperative complications in patients with APGC

	NSR (n = 572)	CR (n = 152)	SPR (n = 113)	*P*
Operation time (min)	192 ± 13	241 ± 21	221 ± 7	< 0.001^a^
Intraoperativetransfusion	154 (26.9%)	68 (44.7%)	36 (31.8%)	< 0.001^b^
Postoperativehospital stay (days)	12.63 ± 1.45	15.25 ± 2.86	12.86 ± 1.68	< 0.001^a^
Examined lymph nodes	25.11 ± 13.71	28.38 ± 14.47	27.02 ± 11.32	0.018^a^
Non-lethal Complication	48 (8.4%)	34 (22.4%)	14 (12.4%)	< 0.001^b^
Anastomotic leakage	14	7	3	
Pancreas-related complications	6	10	0	
Lung pleura- related complications	7	6	2	
Wound complication	5	2	3	
Postoperative ileus	13	6	5	
Liver dysfunction	3	3	0	
Mortality*	3	9	1	NS
Postoperative chemotherapy				0.118
Presence	406(69.4%)	96(16.4%)	83(14.2%)	
Absence	166(65.9%)	56(22.2%)	30(11.9%)	

*NSR*: Nosplenic hilar orsplenic arteryLN resection;*APGC*: Advanced proximal gastric cancer

a: one-way anova,b:χ2test; *:these patients had excluded from the study

## Discussion

The clinical characteristics of patients with PGC and those of patients with gastric cancer located in the distal regions of the stomach differ. For example, patients with PGC have higher LN metastasis and early recurrence rates [12, 13]. Anatomical differences and variations in the lymphatic drainage between patients with PGC and patients with gastric cancers in other locations may lead to higher SLNM rates among patients with PGC. Although the Japanese classification of gastric carcinoma includes the No.10 and No.11 LNs in group 2 of the LNs associated with PGC, many retrospective studies have shown that despite undertaking curative resections, prognosis remains very poor when SLNMs are present. Our results suggest that the 5-year survival rates of patients with SLNMs were 11.4 and 8.1% in the CR and SPR groups, respectively. For such a low survival rate, is dissection of the No.10 and No.11 LNs worth the risk? It is difficult to determine survival significance of the No.10 and No.11 LNs dissections for patients with SLNM because we do not know whether there is metastasis of the No.10 and No.11 LNs in patients with NSR. In this study, we explored the risk factors of SLNM in the CR and SPR groups and distinguished high-risk SLNM patients from NSR patients.

Since spleen-preserving No.10/11 lymphadenectomies may leave residues, we analyzed the characteristics of 152 APGC patients who underwent CR and determined that No.2/4 LN metastases and tumor invasion of the greater curvature were independent risk factors for SLNM. This result is similar to that of previous studies [14, 15]. Moreover, they suggested the existence of an important lymphatic pathway from a primary tumor located in the greater curvature of the stomach to the No.10 or No.11 LNs via the posterior gastric artery, short gastric vessels, or gastroepiploic vessels. This corresponded to observations from a study that used carbon particles to map lymphatic flow in PGC patients [16]. We defined patients with No.2/4 LN metastases and tumor invasion of the greater curvature as being at high risk of SLNMs and verified the reliability of these risk factors in the NSR group. The results showed that patients with high risk of SLNMs had worse prognosis than other NSR patients, and the survival differences were found in the subgroups pT3 and pT4a. This result is similar to that of patients with SLNMs who had worse survival in the CR group and SPR group (Fig. 1b-c, Fig. 2b-c).

Next, the results indicated that patients with SLNMs who underwent No.10 and No.11 LN dissections and those who did not undergo these dissections but were at high risk of SLNM had similar prognosis. These results revealed that splenic hilar or splenic artery LN dissection did not improve survival for patients with SLNMs, whether or not splenectomy or spleen-preserving lymphadenectomy were performed. The poor prognosis of patients with SLNMs might be the same as with patients with distant metastasis and No.10/11 lymphadenectomies have no survival significance. Bian et al. [15] did not recommended the No.10 lymphadenectomy for patients without No.4 s LNs metastasis because they found that patients with negative No.4 LNs who underwent No.10 dissection and those who did not undergo No.10 dissection had similar survival rates. We found similar results (Fig. 3). However, in their study, no survival difference comparison was performed in patients with No.4 LN metastasis. Yang et al. [17] divided patients undergoing total gastrectomy for gastric cancer into two groups, with or without No.10 lymphadenectomy, and no statistically significant difference was found in the 5-year survival rate between the two groups. A limitation of Yang et al.’s study was that there was distributional bias of patients with No.10 LN metastasis in the two groups; for example, there was a greater percentage of patients with No.10 LN metastasis in the No.10 lymphadenectomy group. In our the study, we avoided this bias as well as possible by screening out SLNM high-risk patients from patients without No.10 and No.11 lymphadenectomy.

Finally, we compared the operative features and postoperative complications in the NSR, CR, and SPR groups. The results demonstrated that the NSR group had a shorter operation time, lower incidence of perioperative transfusion, a shorter hospital stay, and lower incidence of non-lethal surgical complications.

This study should be interpreted in the context of its limitations. Although we excluded patients with direct tumor invasion of the spleen or pancreas, there was an unavoidable selection bias in the CR and SPR groups. Patients with more advanced tumors were selected to undergo CR, and patients who were highly suspected of having SLNM were selected to undergo SPR after the mid-1980s. Given the long time period and retrospective nature of this study, there were no standards to guide the lymphadenectomy approach chosen.

## Conclusions

Our findings demonstrated that No.2/4 LN metastases and tumor invasion of the greater curvature were independent risk factors for No.10/11 LN metastases for APGC. Splenic hilar or splenic artery lymphadenectomy with splenectomy or spleen preservation was not associated with increased survival rate of patients with No.10/11 LN metastases.

## Data Availability

The datasets are available from the corresponding authors (Shan Gao, email: mount1121@hotmail.com) on reasonable request.

## Abbreviations

AJCC: American Joint Committee on Cancer
APGC: Advanced proximal gastric cancer
CR: Combined resections
LNs: Lymph nodes
NSR: Spleen-preserving resection
PGC: Proximal gastric cancer
pTNM: Pathological tumor-node-metastasis
SLNMs: Splenic artery lymph node metastases
SPR: Spleen-preserving resection
TNM: Tumor, Node, Metastasis
